# Let-7 microRNA-binding-site polymorphism in the 3′UTR of *KRAS* and colorectal cancer outcome: a systematic review and meta-analysis

**DOI:** 10.1002/cam4.279

**Published:** 2014-06-02

**Authors:** Scott M Langevin, Brock C Christensen

**Affiliations:** 1 Division of Epidemiology and Biostatistics, Department of Environmental Health, University of Cincinnati College of MedicineCincinnati, Ohio; 2 Section of Biostatistics and Epidemiology, Department of Community and Family Medicine, Dartmouth Medical SchoolLebanon, New Hampshire; 3 Department of Pharmacology and Toxicology, Dartmouth Medical SchoolLebanon, New Hampshire

**Keywords:** Cetuximab, lcs6, prognosis, progression, rs61764370, survival

## Abstract

There is a small but growing body of literature regarding the predictive utility of a Let-7 microRNA-binding-site polymorphism in the 3′-untranslated region (UTR) of *KRAS* (*KRAS-LCS6*) for colorectal cancer outcome, although the results are conflicting. We performed a review and meta-analysis in an attempt to better clarify this relationship. A PubMed search was conducted to identify all studies reporting on *KRAS let-7* microRNA*-*binding site polymorphism (*LCS6*; rs61764370) and colorectal cancer outcome. Hazard ratios (HR) and corresponding 95% confidence intervals (CI) were extracted or estimated from each manuscript. Log HRs and log CIs were combined across studies using the inverse-variance weight to calculate fixed- and random-effects summary estimates and corresponding 95% CIs for overall and progression-free survival. We did not observe any significant association between overall or progression-free survival, neither when considering all colorectal cancer patients nor for subgroup analyses (metastatic, anti-EGFR [epidermal growth factor receptor] treatment, or KRAS wild type). There was substantial heterogeneity across studies, overall and among subgroups analyzed. We have found no clear evidence to support an association between the *KRAS-LCS6* genotype and overall or progression-free survival among colorectal cancer patients, even after conducting subgroup analyses by stage and anti-EGFR treatment status. This information helps to clarify the confusing body of literature regarding the clinical implications of the *KRAS-LCS6* genetic variant on colorectal cancer outcomes, indicating that it should not be used at the present time to personalize therapeutic strategies (PROSPERO registration number: CRD42013005325).

## Introduction

It is estimated that colorectal cancer was responsible for over 50,000 deaths in the United States in 2013, making it the second leading cause of cancer-related mortality 1. Despite this, advances in treatment for colorectal cancer have improved disease prognosis over the past decade. Monoclonal antibody therapies that target epidermal growth factor receptor (EGFR), including cetuximab (Erbitux, *ImClone*, LLC) and panitumumab (Vectibix, *Amgen*), are becoming widely used approaches 2, particularly for patients with chemorefractory metastatic disease 3. Through clinical trials, several groups have observed that colorectal cancer patients without somatic mutations in KRAS benefit from anti-EGFR therapy relative to patients harboring a somatic KRAS mutation 4–8. However, only about half of metastatic colorectal cancer patients with wild-type KRAS tumors respond to anti-EGFR treatment 9, indicating a need for additional biomarkers of treatment response. Other prognostic markers have been proposed, including BRAF V600E mutational status 10, but they remain incomplete predictors 11, leaving behind a void in precision medicine therapeutic strategies for colorectal cancer that begs for improvement.

Beyond the use of KRAS mutation status to stratify patients for therapy, normal genetic variation also may contribute to the regulation of *KRAS* and potentially affect response to therapy among patients with wild-type KRAS. For instance, microRNA (miRNA) are known to bind conserved 3′-untranslated regions (UTRs) of genes to prevent their translation, and a single-nucleotide polymorphism (SNP) in the 3′UTR stemming from a T to G transversion in the sixth let-7 complementary site of *KRAS* that affects the binding of let-7 family miRNA and results in lower levels of KRAS expression has been described 12. Since the characterization of the *KRAS* miRNA-related SNP known as *KRAS-LCS6* (rs61764370), there have been a number of studies on the relationship of its genotype with risk 13–18 and prognosis 19–28 of several cancers, with many such studies directed toward colorectal cancer outcome 19–26. To date, studies reporting on the association of *KRAS-LCS6* genotype and colorectal cancer outcome have presented conflicting and clinically confusing results, with some presenting significant estimates with effects in opposing directions. Here, we describe a review and meta-analysis of the relationship between *KRAS-LCS6* genotype with overall and disease-free survival among colorectal cancer patients in an effort to add clarity to the potential implications, if any, of this functional genetic *KRAS* variant on clinical management.

## Methods

### Study identification and selection

Studies reporting on the relationship between the *KRAS let-7* miRNA*-*binding-site polymorphism (*KRAS-LCS6*; rs61764370) and colorectal cancer outcome were identified by entering the following search terms into PubMed (http://www.ncbi.nlm.nih.gov/pubmed): (*“let-7”* OR *let7* OR *lcs6* OR *rs61764370*) AND (*snp* OR *polymorphism* OR *variant*) AND *(colon* OR *colorectal* OR *crc* OR *mcrc*) published in the English language through 31 December 2013. Studies were included if they reported on the *KRAS*-*LCS6* polymorphism and overall or progression-free survival for colorectal cancer patients. The literature was further scrutinized for relevant studies by cross-checking the references of all manuscripts identified through the PubMed search. In the case of overlapping data sets between studies, the most inclusive was retained. This systematic review and meta-analysis was prospectively registered with the PROSPERO database 29 (CRD42013005325).

### Data extraction

Initial eligibility was determined by screening the study abstracts of articles returned in the PubMed search. Articles that were not excluded during the preliminary screening step were examined in more depth by reading the full-text to ensure that they met the inclusion criteria. Study descriptors were derived from the full-text, including the country in which the study was conducted, treatment modalities, stage at diagnosis, primary outcomes, years of recruitment, median age, proportion of male subjects, and races/ethnicities of the study subjects. Additionally, the number and frequency of *KRAS-LCS6* TT versus GT/TT genotype, hazard ratio (HR), and corresponding 95% confidence interval (CI) estimates (or information allowing for the indirect estimation of the HR as described in later sections) were extracted from the full-text. In the absence of adequate information for estimation of a hazard ratio, all efforts were made to contact the authors to obtain sufficient information, as described below.

### Survival curve estimation

Summary survival curves for overall and progression-free survival were estimated by systematically parsing Kaplan–Meier survival curves presented in each manuscript into equal, prespecified, nonoverlapping time intervals (6-month intervals for overall survival; 3-month intervals for progression-free survival) and estimating the survival probability for *KRAS-LCS6* TT and TG/GG genotypes, respectively, using the methods described by Parmar et al. 30. For estimation, censoring was assumed to be noninformative and to have occurred at a constant rate. The number of patients censored at each time interval, *C*_*i*_(*t*_*i*_), was estimated by 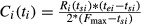, where *R*_*i*_ is the number at-risk, *t*_s_ is the start of the interval, *t*_e_ is the end of the interval, and *F*_max_ is the maximum follow-up in the study. At-risk patients during each interval were calculated as *R*_*i*_(*t*) = *R*_*i*_(*t*_*s*_) − *C*_*i*_(*t*). Summary survival curves were then generated by *KRAS-LCS6* genotype (TT vs. TG/GG) by taking a weighted average based on the number of at-risk subjects for each respective study at each time interval.

### Summary hazard ratio estimates

Log HR and corresponding 95% CI for the association of *KRAS-LCS6* G allele carriers (*G) with overall and progression-free survival were extracted for each study by cancer stage and treatment modality wherever possible. When HR estimates were not provided, they were indirectly estimated by 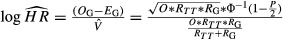, where *O* is the total number of events between both genotype groups, *O*_G_ and *E*_G_ represent the respective observed and expected events for G allele carriers, 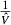 is the estimated Mantel–Haenszel variance of the log-HR, *R_TT_*, and *R_G_*, respectively, represent the total number of patients with *TT* or *G genotype, *p* is the two-sided log-rank *P*-value for a survival difference by *KRAS-LCS6* genotype, and Φ is the cumulative-distribution function for a standard normal distribution. Median survival time is not considered suitable for HR estimation 31.

Log HRs and log CIs were combined across studies using the inverse-variance weight to calculate fixed-effects and random-effects summary estimates (DerSimonian and Laird method) and corresponding 95% CIs 32. In the absence of between-study heterogeneity (*Q*-statistic *P* > 0.05), fixed-effect estimates were reported to conserve statistical power; otherwise random-effects were reported. Meta-analyses were performed for the association of *KRAS-LCS6* *G genotype with overall survival for all studies stratified by cancer stage. Two additional subgroup analyses were performed (1) restricted to metastatic colorectal cancer stratified by treatment modality (anti-EGFR vs. no anti-EGFR) and (2) restricted to KRAS wild-type patients (i.e., without somatic KRAS mutation). Progression-free survival was likewise stratified by treatment modality; a subgroup analysis of KRAS wild-type patients was also performed. Heterogeneity was evaluated quantitatively using the *Q*-statistic and I^2^ metric 33. Risk of publication bias across studies was assessed using the Egger test 34; qualitative likelihood of the summary estimate to be invalidated by bias was also considered.

## Results

### Study selection

**Figure 1:**
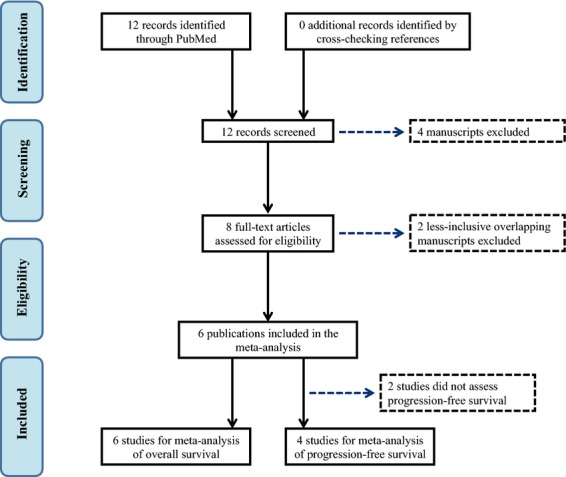
Flow diagram of the study selection for inclusion in the meta-analysis.

The PubMed search returned 12 potential manuscripts 19–26,35–38, of which eight met the inclusion criteria by reporting data on the association between the *KRAS-LCS6* polymorphism and overall and/or progression-free survival for colorectal cancer patients 19–26. Two sets of overlapping study populations were identified 19,21,25,26 among the eight qualified studies, so the less inclusive study for each overlapping was excluded accordingly 21,25. All of the remaining six studies 19,20,22–24,26 reported overall survival and included a total of 1672 patients, while four reported progression-free survival 19,20,23,26 and included a total of 823 patients. A flow diagram of the study identification and selection process is presented in Figure1. Two of the six eligible studies presented results by stage at diagnosis 22,24 (the remaining four studies were restricted to advanced stage metastatic cases) and thus were presented accordingly in the meta-analyses, for a total of nine data sets. Three studies did not report HR 20,23,26, but presented the number of at-risk patients by *KRAS-LCS6* genotype and log-rank *P*-values for survival difference, so additional information (total number of survival and progression events) was obtained through personal communications with the respective authors that allowed for indirect estimation of the HR using the methods described by Parmar et al. 30. The stage-specific number of at-risk patients by *KRAS-LCS6* genotype were obtained for the study by Ryan et al. 22 via personal communication to allow us to use their Kaplan–Meier function for survival curve estimation.

### Study characteristics

A description of the study characteristics for the six eligible studies 19,20,22–24,26 is provided in Table 1. The studies were similar in terms of median age but there was considerable variability with respect to stage at diagnosis and treatment modality. The studies differ by country of origin and there was some variation in terms of racial/ethnic groups included, but the majority of the subjects reported in the literature were white; the study by Ryan et al. 22 was the only publication that contained an appreciable number of non-white patients. Three of the four studies reporting on metastatic colorectal cancer involved only patients treated with anti-EGFR therapy 19,23,26. The study by Kjersem et al. 20 included patients from a randomized clinical trial who were treated with Nordic FLOX (bolus 5-fluorouracil/folinic acid and oxaliplatin) either with or without anti-EGFR therapy and did not present *KRAS-LCS6* survival data by treatment modality. However, we were able to obtain treatment-specific log-rank *P*-values and number of at-risk patients and events to allow for estimation of the treatment-specific HR through personal communications with the authors. Of the four publications that specified treatment with anti-EGFR therapy (cetuximab or panitumumab), all patients included in the studies by Zhang et al. 26 and Sebio et al. 23 were free of any somatic KRAS mutations, while 43% of the patients in Graziano et al. study 19 and 39% of the patients in the Kjersem et al. study 20 harbored a somatic KRAS mutation; the study by Graziano et al. 19 additionally presented a subgroup analysis of 63 KRAS wild-type patients. All of the 121 patients included in the survival analyses by Graziano et al. 19 were free of the BRAF V600E mutation.

**Table 1:** Characteristics of studies included in the meta-analyses

			*KRAS-LCS6* Genotype, *n* (%)									
										
Study	Year	Stage	TT	TG/GG	Country	Patients/treatment	Primary outcomes	Recruitment period	Maximum follow-up (months)	Median age (years)	% Male	Races/ethnicities
Smits et al. 24	2011	Stage I/II	326 (86%)	53 (14%)	Netherlands	Treatment not specified	DS-S^1^	1986–1994	>60	68.0	53%	E
Smits et al. 24	2011	Stage III	137 (81%)	33 (19%)	Netherlands	Treatment not specified	DS-S^1^	1986–1994	>60	67.5	57%	E
Smits et al. 24	2011	Stage IV	54 (78%)	15 (22%)	Netherlands	Treatment not specified	DS-S^1^	1986–1994	52.8^2^	68.5	48%	E
Ryan et al. 22	2012	Stage I/II	88 (83%)	18 (17%)	USA	Treated primarily with 5-FU	OS	1992–2003	>60	n/a	n/a	AA, EA
Ryan et al. 22	2012	Stage III/IV	109 (87%)	16 (13%)	USA	Treated primarily with 5-FU	OS	1992–2003	>60	n/a	n/a	AA, EA
Graziano et al. 19	2010	Stage IV (mCRC)	87 (72%)	34 (28%)	Italy	Chemorefractory mCRC treated with cetuximab + irinotecan	OS, PFS	2005–2008	35.0^2^	65.0	54%	E
Zhang et al. 25	2011	Stage IV (mCRC)	62 (83%)	13 (17%)	USA	Chemorefractory mCRC treated with cetuximab (montherapy)	OS, PFS	2002–2005	18.6^2^	60.0	49%	EA, AA, AsA, O
Kjersem et al. 20	2012	Stage IV (mCRC)	451 (84%)	84 (16%)	Norway	Treated with 5-FU and oxaliplatin ± cetuximab	OS, PFS	2005–2009	47.1^2^	60.4–63.8^3^	60%	E
Sebio et al. 23	2013	Stage IV (mCRC)	72 (78%)	20 (22%)	Spain	Chemorefractory mCRC treated with cetuximab + (irinotecan OR oxaliplatin) or panitumumab (monotherapy)	OS, PFS	2004–2012	43.0	66.0	64%	E

mCRC, metastatic colorectal cancer; 5-FU, 5-fluorouracil; DS-S, disease-specific survival; OS, overall survival; PFS, progression-free survival; E, European; AA, African American; EA, European American; AsA, Asian American; O, other race or ethnicity.

1 Treated as overall survival in the meta-analyses.

2 Approximated from the Kaplan–Meier survivor function provided in the manuscript.

3 Study reports median age by study arm and LCS6 genotype.

### Meta-analyses

#### Overall survival

**Figure 2:**
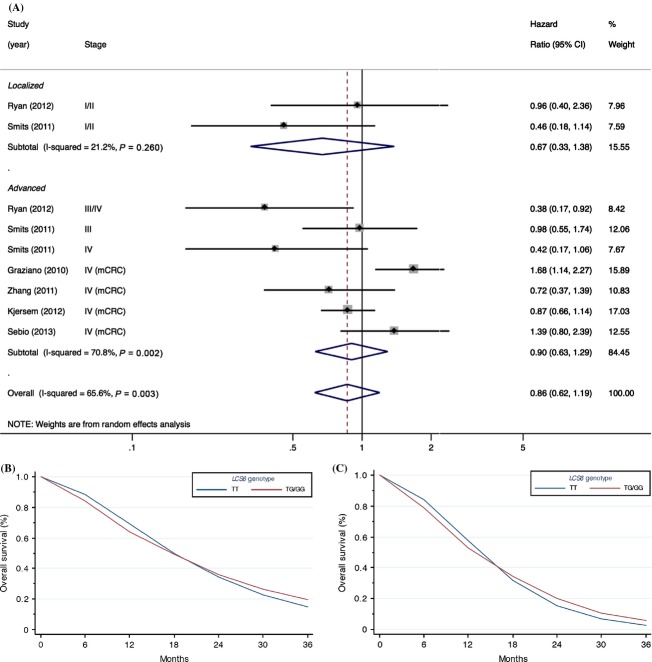
Summary estimates for the association between the *KRAS-LCS6* polymorphism (rs61764370) and overall survival for patients with all stages of colorectal cancer. (A) Forest plot stratified by cancer stage; (B) summary of survival curve for patients with all stages; (C) summary of survival curve for advanced stage colorectal cancer patients. The study by Sebio et al. 23 was not included in the estimation of the meta-survival curve since the manuscript did not present a survival curve. mCRC, metastatic colorectal cancer.

We did not find any relationship between *KRAS-LCS6* genotype and overall survival when considering all colorectal cancer patients regardless of treatment and stage (Fig.2), although there was a moderately large amount of heterogeneity between studies (*P* = 0.003, *I*^2^ = 65.6%). No significant evidence for publication bias was observed using the Egger test (*P* = 0.72), however, it should be noted that there were only nine study estimates included in the test, which can result in low power to detect asymmetry. Likewise, no clear association was present with survival of local (stage I or II) or advanced stage patients (stage III or IV), the latter which had a relatively high degree of heterogeneity between studies (Fig.2).

**Figure 3:**
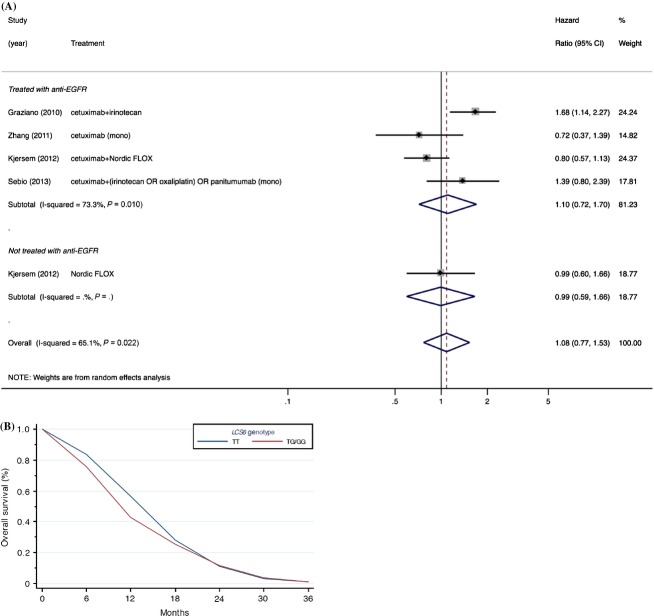
Summary estimates for the association between the *KRAS-LCS6* polymorphism (rs61764370) and overall survival for patients with metastatic colorectal cancer. (A) Forest plot stratified by anti-EGFR treatment status; (B) summary of overall survival curve for patients with metastatic colorectal cancer. The study by Sebio et al. 23 was not included in the estimation of the meta-survival curve since the manuscript did not present a survival curve. EGFR, epidermal growth factor receptor; mono, monotherapy; Nordic FLOX, bolus 5-fluorouracil/folinic acid and oxaliplatin.

Since *LCS6* is a functional SNP in the *Let-7*-binding site of *KRAS* that impacts KRAS expression, and KRAS overexpression has been accepted in clinical practice as a negative predictive biomarker for patients treated with anti-EGFR therapy 39, we performed a subgroup analysis on metastatic colorectal cancer patients treated with anti-EGFR therapies (four studies with an aggregate of 643 patients). However, despite the reported potential of the *LCS6* SNP to elevate KRAS expression 14, no association was observed (Fig.3). Contrary to our efforts to reduce between-study heterogeneity through subgroup analysis restricted to metastatic colorectal cancer patients with metastatic disease treated with anti-EGFR therapies, a high degree of heterogeneity remained (*P* = 0.01, *I*^2^ = 73.3%). Likewise, no association was observed after further restriction to KRAS wild-type metastatic colorectal cancer patients (Fig. S1); albeit somewhat attenuated, a moderate degree of heterogeneity remained between studies (*I*^2^ = 44.0%).

#### Progression-free survival

**Figure 4:**
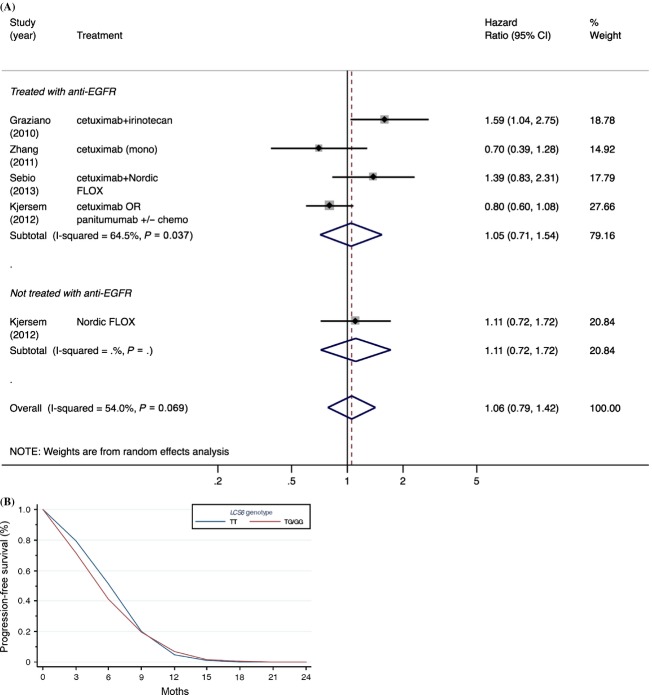
Summary estimates for the association between the *KRAS-LCS6* polymorphism (rs61764370) and progression-free survival for patients with metastatic colorectal cancer. (A) Forest plot stratified by anti-EGFR treatment status; (B) summary of progression-free survival curve for patients with metastatic colorectal cancer. The study by Sebio et al. 23 was not included in the estimation of the meta-survival curve since the manuscript did not present a survival curve. EGFR, epidermal growth factor receptor; mono, monotherapy; Nordic FLOX, bolus 5-fluorouracil/folinic acid and oxaliplatin.

We also found no significant association between *KRAS-LCS6* genotype and progression-free survival (Fig.4), which was reported only by the four studies that restricted enrollment to metastatic colorectal patients (with an aggregate of 672 patients) 19,20,23,26, with a moderate degree of heterogeneity between studies (*P* = 0.07, *I*^2^ = 54.0%). No significant evidence for publication bias was observed using the Egger test (*P* = 0.24), although this should be interpreted with caution since there were only five study estimates included in the test (separate HR estimates were included in the meta-analysis by anti-EFGR treatment status for the study by Kjersem et al. 20), which could adversely impact power to detect asymmetry. No significant association was observed in the subgroup analysis restricted to KRAS wild-type patients (Fig. S2).

## Discussion

After reviewing and summarizing the literature, we found no clear association between the *KRAS-LCS6* genotype and overall or progression-free survival among colorectal cancer patients, even after conducting subgroup analysis by stage and anti-EGFR treatment status. These results suggest that *KRAS-LCS6* genotype is an insufficient predictor of outcome by itself and they provide insight into the conflicting body of literature surrounding clinical utility of *KRAS-LCS6* genetic testing in the clinical management of this disease, demonstrating the complexity of colorectal cancer and the need for additional, more complex batteries of molecular markers to optimize therapeutic regimens guided by precision medicine approaches.

Notwithstanding our best efforts to reduce between-study heterogeneity through subgroup analyses, a substantial amount of heterogeneity remained. The prospective nature of the studies included in the meta-analysis reduces the risk of bias among the individual studies and no publication bias was observed across studies. However, even after restricting to studies of metastatic colorectal cancer patients treated with anti-EGFR therapies, heterogeneity remained, suggesting a possible important role of concomitant treatments. Although these four studies 19,20,23,26 had in common the use of anti-EGFR therapy, most commonly cetuximab, one study also included patients treated with panitumumab. Furthermore, not all studies were restricted to patients lacking somatic KRAS or BRAF mutations, and there was broad variability in terms of combination therapy used (if any), including anti-metabolites (5-fluorouracil), platinum-based cross-linking agents (oxaliplatin), or topoisomerase inhibitors (irinotecan). This indicates a need for additional in-depth treatment-modality-specific research into the impact of this disease in patients free of somatic KRAS and BRAF mutations.

Strengths of this meta-analysis include the prospective nature of the reviewed studies and our ability to discriminate by stage at diagnosis, including patients with advanced metastatic disease. The aggregate nature of this meta-analysis, which greatly enhanced the sample size in our analyses, is another major strength of this study. Post hoc calculations indicate that we had ample statistical power to detect clinically relevant associations, with ≥80% power to detect an HR >1.19 and 1.22 for our overall and progression-free survival, respectively, and ≥1.26 for each respective outcome in our analyses that were restricted to anti-EGFR treated patients. Furthermore, our adherence to the PRISMA statement guidelines 40 underscore the systematic nature of our comprehensive review and meta-analysis and enhance the transparency of our methods and results. Additionally, we were able to estimate survival curves for all but one of the studies included in our meta-analysis, providing a visual companion to the summary hazard ratio estimates allowing for better interpretation of the results. However, there were also several limitations to the study. Hazard ratio estimates were only directly available for three of the six studies included in the meta-analyses and therefore indirect estimation methods had to be applied, which may not exactly reflect the true measured effect, although it is doubtfully based on the data that this could have impacted the overall lack of significant associations observed in this meta-analysis. Furthermore, as is the case with most meta-analyses, the summary estimates are based on aggregate results in published literature, rather than individual-level patient data, which could potentially introduce bias. However, in order for confounding to impact study estimates, *KRAS-LCS6* genotype would have to be associated with another unaccounted factor that also impacts prognosis, which although conceptually plausible, is unlikely. Additionally, as the patients included in the published studies were predominantly Caucasian, which is the population with the highest variant allele frequency (∼0.15) based on estimates provided by dbSNP (http://www.ncbi.nlm.nih.gov/SNP/) and Allele Frequency Database (ALFRED; http://alfred.med.yale.edu/), it is unclear how these findings generalize to patients of other races/ethnicities, among whom the variant allele is much less common.

Our results show that *KRAS-LCS6* genotype alone is not a meaningful predictor of outcome for colorectal cancer patients as a whole or for those with metastatic disease treated with anti-EGFR therapy. However, as several of the studies included in this review reported significant associations with outcome in well-conducted, prospective studies, it arouses the possibility that the prognostic value of *KRAS-LCS6* genotype may be largely dependent on the combination therapy (if any) used in conjunction with the anti-EGFR treatment. Additional future studies are required to determine the effectiveness of *KRAS-LCS6* genotype in the prognostication of patients treated with specific anti-EGFR mono and combination therapy regimens.

## Supporting Information

**Figure S1.** Forest plots for the subgroup analyses of the association between *KRAS-LCS6* and overall survival for patients lacking a somatic KRAS mutation. **Figure S2.** Forest plots for the subgroup analyses of the association between *KRAS-LCS6* and progression-free survival for patients lacking a somatic KRAS mutation.
