# Supplementary material for: Let-7 microRNA-binding-site polymorphism in the 3′UTR of KRAS and colorectal cancer outcome: a systematic review and meta-analysis
Source: Cancer Med. 2014 Jun 2;3(5):1385–95. doi: 10.1002/cam4.279 (PMC4302689; doi:10.1002/cam4.279)

Figure S1. Forest plots for the subgroup analyses of the association between *KRAS-LCS6* and overall survival for patients lacking a somatic KRAS mutation.

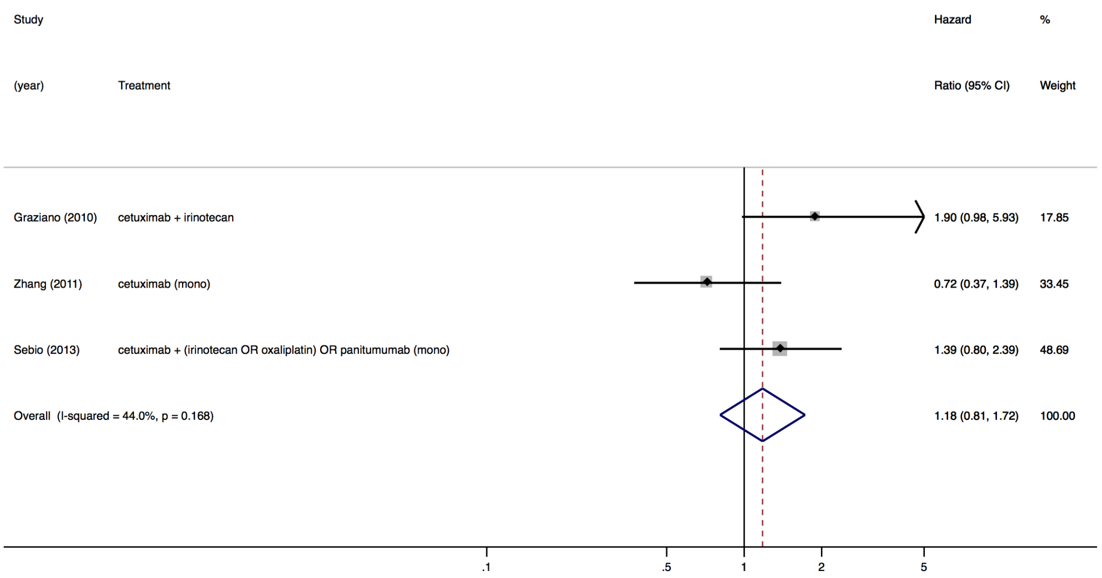

Figure S2. Forest plots for the subgroup analyses of the association between *KRAS-LCS6* and progression-free survival for patients lacking a somatic KRAS mutation.

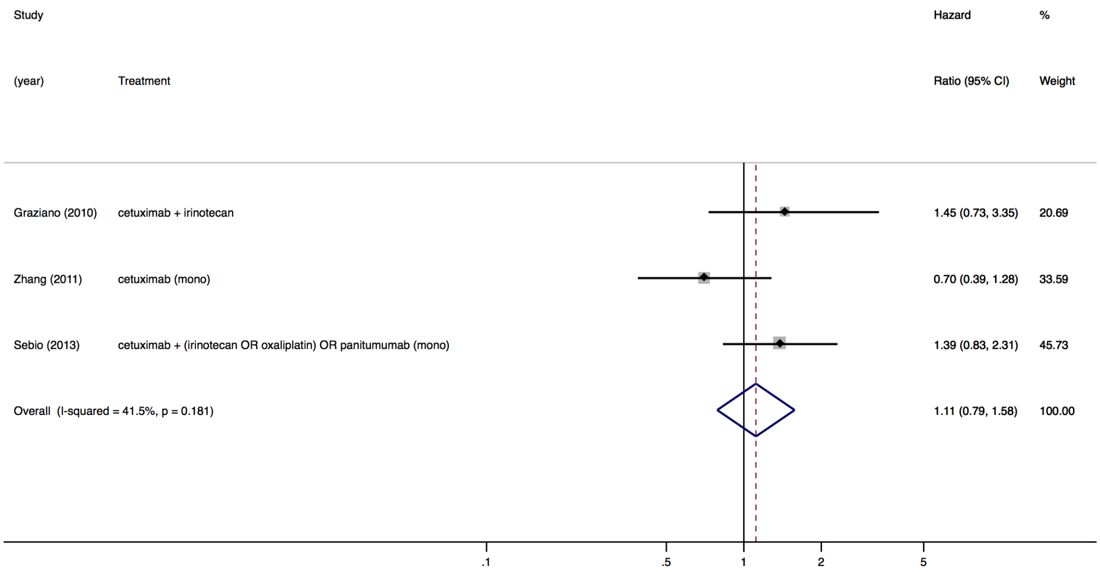

Supplement: Supplementary file 1 [file cam40003-1385-SD1.pdf]
